# A Multiobjective Optimization Model for a Dynamic and Sustainable Cellular Manufacturing System under Uncertainty

**DOI:** 10.1155/2022/1334081

**Published:** 2022-09-22

**Authors:** Javad Jafarzadeh, Hossein Amoozad Khalili, Naghi Shoja

**Affiliations:** ^1^Department of Industrial Engineering, Science and Research Branch, Islamic Azad University, Tehran, Iran; ^2^Department of Industrial Engineering, Sari Branch, Islamic Azad University, Sari, Iran; ^3^Department of Mathematics, Firoozkooh Branch, Islamic Azad University, Firoozkooh, Iran

## Abstract

For many years, cellular manufacturing has been implemented by owners of manufacturing units. Furthermore, the increasing importance of sustainable development has led manufacturers and managers to consider the concepts of sustainable manufacturing. Sustainable manufacturing includes three components: economic, environmental, and social responsibility. Many research and studies have been conducted in the field of cellular manufacturing, and also in most studies, only the economic component or at most two components of sustainable manufacturing have been taken into account. With increasing concerns about global warming, environmental issues have become particularly important in the production of products and goods. On the other hand, customer satisfaction as one of the aspects of social responsibility is of significant importance. In this research, we put the sustainable manufacturing system in the dynamic cellular manufacturing system under uncertainty (fuzzy parameters). A multiobjective sustainable mathematical model with objective functions of minimizing costs minimizing CO_2_ emissions and minimizing product shortages (customer satisfaction) was proposed. In order to confirm the validity and accuracy of the proposed model, a small example was solved in GAMS software with CPLEX solver and epsilon constraint method, and its basic variables were investigated. Then, due to the high complexity of the proposed cellular manufacturing model, two meta-heuristic algorithms NSGA-II and MOGWO were used to solve larger problems in MATLAB software. To compare the performance of the two proposed algorithms, ten problems with different dimensions were designed and then the two algorithms were compared with each other based on several performance evaluation indicators. Also, in order to investigate the significance of the difference between the two algorithms based on each index, a statistical analysis was carried out by Minitab software. Taguchi method was also used to adjust the parameters of both algorithms. Based on the analytic results and statistical analysis, the MOGWO algorithm performed better than NSGA-II algorithm and the exact solution method, GAMS software.

## 1. Introduction

Dynamic cellular manufacturing is one of the newest manufacturing concepts that has been regarded in recent decades. The advantages of implementing it include in increasing the efficiency and productivity of workforce and space, reducing costs, reducing inventory while manufacturing and reducing costs, and improving manufacturing planning. Sustainable manufacturing has recently been considered by the owners of manufacturing firms. The benefits of its implementation include improving the morale and health of employees, employee responsibility and environmental protection, reducing pollution, and increasing profitability [1]. Considering the importance of the two mentioned issues, methods have been proposed to integrate the two.

The growing awareness of environmental protection has highly affected the production methods of manufactured products. Introduction of new methods demonstrates the features and advantages of sustainable manufacturing management over conventional and traditional methods. Sustainable manufacturing systems have attracted a great deal of attention in the last 20 years (as an emerging manufacturing approach). Especially in the last 10 years, the number of papers focusing on the management of sustainable production systems has increased rapidly. There are more practical and integrated factors taken into account that make it more complex but closer to reality. Sustainable manufacturing is the creation of products produced through economic processes that minimize negative environmental impacts, while saving energy and natural resources, as well as increasing employee satisfaction, and community safety and product health [2].

Cellular manufacturing systems are among the new manufacturing methods that are used today in most of large manufacturing centers with high product diversity and multipurpose facilities. The basis of a cellular manufacturing system is the classification of products and machines based on their physical, operational, or processing similarity into several smaller manufacturing units called cells. Cells can be physical or virtual in nature. On the other hand, in most manufacturing units, the traditional perspective is used for supply and distribution planning. This means that each of these components independently plans for its activities. The supply chain in the manufacturing network seeks to create coordination between manufacturing facilities, suppliers, and allocation of products to product markets. Most manufacturing companies first design the supply chain and the number and locations of manufacturing facilities and make decisions about which product markets each facility serves. Then, they organize the processes (production line, cell arrangement, etc.) in each facility. In this case, the cellular arrangement will be done without considering the manufacturing network and chain. Simultaneously addressing the supply chain design and factory cellular manufacturing design will lead to the design of a manufacturing network with lower production and distribution costs to respond quickly to customers in return for having fewer facilities in manufacturing products. Furthermore, under real condition, the nature of many manufacturing parameters such as product demand, available machine capacity, and processing time are uncertain. Therefore, it is difficult to determine the cellular arrangement, the amount of manufacturing, and the amount of purchase according to the demand of customers' products in a competitive market that is facing uncertainty. Cellular manufacturing system is one of the new manufacturing methods that, in recent years, the industrial sector has benefited from its advantages, including reducing the amount and cost of transportation, reducing commissioning time, reducing manufacturing time, reducing package size, and reducing inventory during manufacturing.

Today, due to being in the movement of global competitive markets, manufacturing factories that are characterized by the specialization of needs, shortening of the product life cycle, shortening of the product supply cycle to the market and the various demands of customers should adapt to these conditions, and take measures that improve the efficiency and productivity of manufacturing processes [3]. Therefore, a mixed manufacturing system called dynamic cellular manufacturing was developed. This research has been conducted in order to achieve the main objective of integrating the problem of cell manufacturing and sustainable manufacturing by considering uncertainty in the safe scaffolding manufacturing industry and solving the proposed problem. Also, the subobjectives of the present study include considering environmental issues in the problem of cellular manufacturing, including the concept of social responsibility as one of the dimensions of sustainability in the problem of sustainable manufacturing and solving the problem of cellular manufacturing in conditions of uncertainty.

As mentioned, sustainable manufacturing has social, economic, and environmental aspects. The introduction of these concepts in industries and manufacturing will increase the efficiency and prosperity of manufacturing and respect for human issues psychologically and physically and respect for the environment in all its aspects and further progress in economic concepts. For example, in this study, an example of safe scaffolding manufacturing is given, which we do not enjoy its benefits in the country due to the lack of knowledge that is the manifestation of three criteria of sustainable manufacturing. Annually, a large number of workers lose their lives or become disabled due to noncompliance with safety issues. The urban environment is unbeautiful and unsafe because of old scaffolding. More iron is used to set up old scaffolding. This example alone illustrates the need for research in this area. And on the other hand, the benefits of cellular manufacturing mentioned earlier and considering both as integrated multiplies the importance of the issue. Even in industrialized countries, concerns about the issue of environmental pollution and greenhouse gases and other environmental issues and concerns are increasing every day. Social debates and not considering them in manufacturing decisions cause irreparable damage to society. All this increases the need for research on this subject.

Integration of dynamic cellular manufacturing systems by considering the concepts of sustainable manufacturing (economic, environmental, and social responsibility) in uncertainty is one of the innovations of this research. In this regard, we will try to provide multiobjective mathematical models to design a group arrangement in (1) formation of dynamic cells with the aim of decreasing the corresponding cost, (2) reduction in cost and time related to the concepts of sustainable manufacturing in the dynamic cellular manufacturing system and increasing the system efficiency in the real world in the manufacturing of safe scaffolding. Providing two meta-heuristic approaches in order to solving the proposed models, in this regard, we will also try to use meta-heuristic approaches in order to solve the proposed models. This research could be used in the future by researchers and students studying dynamic cellular manufacturing systems. One of the innovations of this research goes back to the fact that in addition to the usual research that is generally conducted in the field of dynamic cellular manufacturing systems, in this dissertation, sustainable manufacturing is also integrated with this issue, and three dimensions of sustainability (economic, environmental, and social responsibility) are used in this issue. Also, due to the consideration of uncertainty (fuzzy data), the manufacturing environment is considered to be more realistic, and also meta-heuristic methods are used to solve the proposed problem. Furthermore, the implementation of this issue in the safe scaffolding manufacturing industry is another highlight of this research. Having an integrated view of the above points is an issue that is of great importance for the reasons mentioned. This is one of the research gaps (especially in the real world and especially scaffolding manufacturing). In other words, no research has been conducted in the country considering three dimensions of sustainable manufacturing simultaneously.

The rest of this research is organized in this way. In the second section, an introduction to the research literature and the history of the subject will be given. In the third section, while stating the proposed model, in order to confirm the validity and accuracy of the mathematical model, a problem with small dimensions in the safe scaffolding manufacturing industry is solved by the epsilon constraint method in GAMS software, and the basic variables are examined. In the fourth section, due to the NP-Hard nature of the proposed problem, two algorithms of NSGA-II and MOGWO are used to solve the mathematical model. Finally, conclusion and future suggestions are provided.

## 2. Literature Review

In this section, we describe the studies that have been carried out so far in the fields of cellular manufacturing, design of dynamic cellular manufacturing systems and cellular configuration and arrangement of cellular machines in dynamic conditions and sustainable manufacturing and (fuzzy) uncertainty.

Achieving a cellular manufacturing system requires three fundamental changes: a change in the arrangement and organization of machines and the formation of manufacturing cells, a change in the material ordering system and inventory control, and a change in the production planning system.

Saidi-Mehrabad and Safaei [4] proposed a comprehensive mathematical model with dynamic cellular configuration, alternative routing, part distribution, operation sequence, multiple units of identical machines, machine capacity, workload balance between cells, operation cost, subcontracting cost, cost of used tools, cost of preparation, size of cells, and proximity constraint of machines. Safaei and Tavakoli Moghaddam [5] assume a developed model in which parts in packets are moved between cells with assumptions such as plan of alternative operations, sequence of operations, capacity of machines, and capability multiplication of machines. The purpose of their model was to minimize the sum of the fixed and variable costs of machines, intercellular movement, and reconfiguration. Ah Kioon et al. [6] combined production planning and cellular manufacturing system in their research to minimize the total costs of intercellular material movement, storage of final items, preparation of the system for processing different parts in different time periods, and performing operations by machines.

Kia et al. [7] in their research described the problem of dynamic cellular arrangement using multiple conflicting goals. This paper presents the multiobjective nonlinear mathematical model on the dynamic cell formation (DCF) problem by weighting three conflicting objectives including the cost of moving machines in the cellular rearrangement process, the rate of utilization of machine capacity, and the total number of intracellular movements across the entire planning horizon. The model is solved by CPLEX, and because it is time consuming, the problem is solved using the scatter search algorithm. The results show the effectiveness of the scatter search algorithm. Rabbani et al. [8] considered the problem of designing cellular manufacturing systems in dynamic environments with changes in machine reliability and part demand. This paper proposes a multiobjective integer programming model for multiple planning horizons with the aim of maximizing machine reliability and minimizing system cost. The proposed model is solved using the branch and bound method and Lingo software.

Ahiska and Kurtul [9] in their study measured the performance of sustainable manufacturing with presence of recyclable waste, in which they used the two-stage data envelopment analysis method. Moldavska [10] expresses the evaluation of the model based on sustainable manufacturing. This paper examines the potential of studying a sustainable economy. The sustainable manufacturing model is based on the ideas of complexity theory. The development of the sustainable development index for the manufacturing industry is considered in this study; the definition of sustainability is clear: manufacturing is positive and at the same time has little effect on inflexible resources. The purpose of this study is to determine the sustainability index of the manufacturing plant. Since the topic is so wide-ranging, this research is limited to small and medium-sized industries with defined set of joint operations and process plans.

Ma et al. [11] reviewed more than 100 related papers, mainly 1994–2015, in the field of classification of mathematical problems with the management of sustainable manufacturing systems and divided them into three categories according to the main elements in a manufacturing system: production planning and control, inventory management, and control and building of network design. A major challenge for manufacturers is not only designing but also producing products using a sustainable approach. Sustainable development consists of three structural pillars: society, environment, and economy but meanwhile includes operational aspects such as resource consumption, natural environment, economic performance, workers, products, social justice, and society development. Manufacturing industries have recognized that their task is to design a sustainable manufacturing system that has fewer environmental impacts and social disruptions and promotes wealth. One of the key results in this study is that technology capability and economic risk are the two main factors that prevent a company from making a decision to implement sustainable manufacturing.

Singh et al. [12] in their study provided a specialized system based on fuzzy law to evaluate the sustainable performance of manufacturing in small and medium enterprises. The initial set of measures and measurements were determined based on their characteristics. Sixteen criteria were identified and classified under four economic measures, five environmental measures, and three social measures.

Koren et al. [13] stated 6R (reduction, reuse, recycle, recover, redesign, and remanufacturing) in their research and considered it as a basis for the factory to manufacture the next generation, which can significantly increase system sustainability, quickly adjust system configuration and production processes to respond to emerging market demand, and maintain system values for future products. Mutingi et al. [14] used the modular production design approach for sustainable manufacturing in the fuzzy environment. Salido et al. [15] stated in their research that manufacturing industries face environmental challenges; therefore, their industrial processes should be optimized in terms of profitability and sustainability. Since many of these processes are dynamic, this paper addresses the improvement in dynamic planning problems in workshop manufacturing where machines can operate at different rates. The results provided in this paper may be useful for use in real industries to redesign cost-effective manufacturing.

Singh and Sultan [16] in their research provided a model using the theory of complexity to evaluate sustainability in sustainable manufacturing. Fisher et al. [17] introduce research in the field of cloud manufacturing as a future for application in sustainable manufacturing. In their research, they introduced four methods to increase sustainability, including calibrating the design and increasing automation.

Peng et al. [18] provide a paper to improve the manufacturing sustainability of workshop machinery in workshop manufacturing. This paper provides a common multiobjective model of energy consumption and production efficiency. Moldavska and Martinsen [19] stated in their research that the number of scientific papers in the field of sustainable manufacturing shows a significant growth in interest in this subject in the last 20 years. Contrary to many published papers, the deep purpose of sustainable manufacturing or at least a support of a strong theory is still missing. 6R is the first attempt to resolve this issue. In this study, they sought to expand this concept.

Malek and Desai [20] stated that sustainable manufacturing is a combination of the triple bottom approach (economic, environmental, and social) from manufacturing methods. In such a complex system, decision-making becomes difficult in terms of selecting and prioritizing various aspects. Multiple multicriteria decision-making methods can facilitate selection and prioritization in a complex system. Their research prioritizes sustainable manufacturing barriers by calculating weight by using the best and worst methods in an Indian manufacturing organization. Beekaroo et al. [21] in a study entitled development of a sustainability index for Mauritian manufacturing companies examined the sustainability indices in 30 Mauritian companies in terms of nine environmental indices, four economic indices and two social indices.

Zheng et al. [22] in their research addressing to energy saving and pollution reduction by reducing production deficits. While previous research has focused on reducing time and labor. Pagone et al. [23] discuss the concept of decision-making in the selection of parts according to sustainability criteria, in which 18 criteria are divided into four groups of time, cost, quality, and environment, and through TOPSIS, they concluded that aluminum alloy would be the best choice for manufacturing automobile parts.

Pourghader Chobar et al. [24] proposed a multiobjective location-routing problem model for multidevice relief logistics under uncertainty using meta-heuristic algorithm. Rezaei Kallaj et al. [25] presented a research in which the problem of vehicle routing in relief supply under a crisis condition considering blood supply has been presented. Pourghader Chobar et al. [26] proposed a multiobjective model for hub location problem considering dynamic demand and environmental issues.

Table 1 summarizes the comparison of research conducted regarding the subject literature, based on which research gaps are explored.

In order to take advantage of the benefits of dynamic cellular manufacturing and sustainable manufacturing, which were briefly mentioned above, we need to consider these two concepts integrated. The research gap that is the reason for conducting this research is also this issue that as mentioned in the tables above has received little attention. In this research, we put the dynamic and sustainable cellular manufacturing system in conditions of uncertainty. Operators have not been considered by researchers in previous research, while they are the basis for implementing sustainable manufacturing. The main aspects of sustainability are economic, environmental, and social responsibility, which in this study are considered as objective functions of the proposed mathematical model.

## 3. Problem Definition and Mathematical Modeling

In this section, according to the research gaps mentioned in the previous section, the place of the forthcoming research was determined, followed by the definition of the problem and the description of the research assumptions in such a way that it can cover the shortcomings of the study area as much as possible. First, the fuzzy mathematical model is provided and then its defuzzification method is explained, and the defuzzificated model is provided. Finally, an example in small dimensions is solved in GAMS software by CPLEX solver and epsilon constraint method to validate the proposed model and the results are expressed.

Cellular manufacturing systems are among the new manufacturing methods that are used today in most of large manufacturing centers with high product diversity and multipurpose facilities. The basis of a cellular manufacturing system is the classification of products and machines based on their physical, operational, or processing similarity into several smaller manufacturing units called cells. Cells can be physical or virtual in nature. On the other hand, in most manufacturing units, the traditional perspective is used for supply and distribution planning. This means that each of these components independently plans for its activities. Today, due to being in the movement of global competitive markets, manufacturing factories that are characterized by the specialization of needs, shortening of the product life cycle, shortening of the product supply cycle to the market, and the various demands of customers should adapt to these conditions and take measures that improve the efficiency and productivity of manufacturing processes. Therefore, a mixed manufacturing system called dynamic cellular manufacturing was developed. Sustainable manufacturing has economic, social, and environmental dimensions. Sustainable manufacturing is the creation of products produced through economic processes that minimize negative environmental impacts, while saving energy and natural resources, as well as increasing employee satisfaction, and society safety and product health.

### 3.1. Mathematical Modeling

The assumptions of the problem are as follows:

1. Shortage is permissible and limited.
2. Each machine has a fixed cost as an overhead cost and is not related to its consumption.
3. The variable cost of each machine depends on its working hours.
4. If a machine is purchased, it must be in the workshop in the next time periods and the removal of the machine is not considered.
5. Partitioning between cells is not considered and one place can be assigned to different cells.
6. Moving machines includes installation and removal. When a machine is moved from one place to another, both installation and removal costs are covered. Also, regardless of the cost of purchasing a machine, the installation cost is considered for the machines.
7. Intracellular and extracellular transmission costs of products are linear function of distance.
8. For the number of machines assigned to each cell, an upper and lower limit is considered. The large number of machines in each cell complicates the production control in the cell. On the other hand, the small number of machines in the cell increases the intercellular transmission costs.
9. The shape and position of the cells are not predetermined.
10. Operators based on their expertise can operate on the machines in question and there is no limit to the number of operators.

In this section, the desired fuzzy mathematical model is proposed. The proposed mathematical model has three objective functions. The constraints and objective functions and a brief description of them are provided below.

#### 3.1.1. Sets

 
*p*: set of product types *p*{1,2,…, *P*}
 
*t*: set of time periods *t*{1,2,…, *T*}
 
*r*: set of operations required to perform on products *r*{1,2,…, *R*_*p*_}
 
$c,\overset{´}{c}$: set of cells *c*{1,2,…, *C*}
 
$l,\overset{´}{l}$: set of places for establishment of machines *l*{1,2,2 …, *L*}
 
$w,\overset{´}{w}$: set of operators based on expertise *w*{1,2,…, *W*}

#### 3.1.2. Parameters

 
${\tilde{CI}}_{p}$: intracellular transmission cost per unit of product *p* per unit of distance
 
${\tilde{CE}}_{p}$: intercellular transmission cost per unit of product *p* per unit of distance
 
${\tilde{d}}_{pt}$: demand for product *p* at time *t*
 
$Q_{l\overset{´}{l}}$: distance between places *l* and *l*′
 
${\tilde{CINS}}_{m}$: installation cost of machine type *m*
 
${\tilde{CPUT}}_{m}$: cost of removing machine type *m*
 
${\tilde{CF}}_{m}$: overhead cost of machine type *m* in each time period
 
*CV*_*m*_: variable costs of machine type *m*
 
*CB*_*m*_: cost of purchasing machine type *m*
 
*Ucap*_*c*_: maximum number of machines to be assigned to cell *c*.
 
*Lcap*_*c*_: minimum number of machines to be assigned to cell *c*.
 
*A*_*m*_: area of machine type *m*
 
${\tilde{\omega }}_{prm}$: processing time of the *r*th operation for product *p* on machine type m
 
${\tilde{cap}}_{m}$: time capacity of machine type *m* in each time period
 
$\tilde{Tcap}$: total capacity of the workshop to store products
 
*Gcap*: maximum limit for carbon dioxide emissions
 
*V*_*p*_: volume of product *p*
 
${\tilde{CO}}_{w}$: cost of each working day of the operator with expertise in *w*
 
*g*_*pm*_: Co_2_ emission rate due to the production of each unit of product *p* on machine type m
 
${\tilde{CH}}_{p}$: inventory cost per unit of product *p*
 
*SS*_*p*_: maximum shortage product of *p* in each period

#### 3.1.3. Decision Variables

 
*x*_*clmwt*_: if the operator with expertise *w* is assigned to machine type *m* in place *l* in cell *c* at time *t* it is 1, otherwise 0.
 
*y*_*prmw*_: if the *r*th operation of product *p* can be performed on machine type *m* by the operator with expertise *w* it is 1, otherwise 0.
 
*S*_*pt*_: shortage of product *p* at time *t*
 
*I*_*pt*_: amount of product *p* at time *t*
 
*u*_*prclmtw*_: number of p-type products on which the *r*th operation was performed by machine type *m* in place *l* in cell *c* at time *t* by the operator with expertise *w*.
 
$NP_{prc\overset{´}{c}l\overset{´}{l}m\overset{´}{m}t}$: number of *p*-type products that were processed at time *t* by the *r*th operation on machine type *m* in place *l* in cell *c*, and now they were transferred on machine *m*′ in cell *c*′ in place *l*′ to perform the *r* + 1th operation.
 
*v*_*lmt*_: if place *l* is empty at time t − 1 or assigned to a machine other than machine *m* and assigned to machine *m* at time *t* it is 1, otherwise 0.
 
*d*_*lmt*_: if machine type *m* is assigned to place *l* at time *t* − 1 and removed at time *t* or replaced by another machine it is 1, otherwise 0.
 
*N*_*mt*_: total number of *m*-type machines that are added to the workshop level at the beginning of period *t*.
 
*NO*_*wt*_: number of operators available with expertise *w* at the workshop level at time *t*.

Before providing the mathematical model of the problem, since some of the parameters of the problem are considered uncertain, we introduce the possibilistic programming model and then express the final uncertain mathematical model that is modeled using the linear possibilistic programming.

Philosophically, fuzzy logic expresses a concept of logic in which, unlike Aristotelian logic, which rules classical mathematics, it uses multivalued logic to express its concepts. This logic, unlike Aristotelian logic, which always considers everything as being or not being, does not assume a boundary between being and not being. In this logic, anything can belong to a group to a certain extent. The important point from our point of view in this dissertation is the application of fuzzy models on the subject of this dissertation. The complex nature of DSCM makes its mathematical model parameters uncertain. As mentioned, there are several types of uncertainty, one of which is possibilistic programming uncertainty, which can be cited when there is insufficient information about the parameters. And since the parameters and information about the DCMS problem are uncertain, fuzzy logic is used for the input information. The research method of [29] is used to convert the possibilistic model of the problem, which includes inaccurate coefficients in both the objective function and the constraints, into a certain model. This method is computationally efficient because it retains the linearity property and also does not increase the number of objective functions and unequal constraints. Due to the simplicity of obtaining the data, the triangular fuzzy distribution is used to model the inaccurate nature of the ambiguous parameters of the problem. Assume that $\tilde{c}=\left(c^{\left(1\right)},c^{\left(2\right)},c^{\left(3\right)}\right)$ is a triangular fuzzy number and the following model is considered:

(1) $$\begin{array}{c} \text{Min }Z=cx\\ \text{s.t}:Ax\leq b\\ Dx\;\geq f\\ x\geq 0. \end{array}$$

Now, assuming that *x* is the decision variable and *c*, *b*, *A*, *D*, and *f* are the model parameters and the parameters *c*, *b*, and *f* are fuzzy, then the defuzzificated model is as follows:

(2) $$\begin{array}{c} \text{Min }Z=\;\left[\frac{c^{\left(1\right)}+c^{\left(2\right)}+c^{\left(3\right)}}{3}\right]x\\ \text{s.t}:Ax\leq \left[\left(2\alpha -1\right)b^{\left(1\right)}+\left(2-2\alpha \right)b^{\left(2\right)}\right]\\ Dx\;\geq \left[\left(2\alpha -1\right)f^{\left(3\right)}+\left(2-2\alpha \right)f^{\left(2\right)}\right]\\ x\geq 0. \end{array}$$

Defuzzification is from the Fazle method. *C*, *b*, and *f* are uncertain and have a triangular membership function, in the objective function, the average of the parameter is uncertain and in the constraint of greater than or equal to like demand, the criterion for satisfying demand is the larger number, and in the constraint of smaller than or equal to like capacity, the criterion is the smaller number. Just before each fuzzy parameter in the constraint proportional to the larger and smaller inequality, according to Relation (2), there is an expression that by changing the coefficient *α*, which is actually the level of confidence, sensitivity analysis is carried out on fuzzy parameters. The higher this coefficient becomes and gets closer from 0.5 to 1, the more number is multiplied by the pessimistic number and the closer it gets to 0.5, the more value is multiplied by the possible number.

The parameter *α* must take a value in the range of 0.5 to 1. Based on the method of [29].

The final uncertain model:

(3) $$\begin{array}{c} \text{Min }Z_{1}=\sum_{t,p,r,c,\overset{´}{c},l,\overset{´}{l},m,\overset{´}{m}}U_{prc\overset{´}{c}l\overset{´}{l}m\overset{´}{m}w\overset{´}{w}t}\times Q_{l\overset{´}{l}}\times \left[\frac{CE_{p}^{1}+CE_{p}^{2}+CE_{p}^{3}}{3}\right]\\ +\;\sum_{t=2,l,m}v_{lmt}\times \left[\frac{CINS_{m}^{1}+CINS_{m}^{2}+CINS_{m}^{3}}{3}\right]\\ +\;\sum_{t=2,l,m}d_{lmt}\times \left[\frac{CPUT_{m}^{1}+CPUT_{m}^{2}+CPUT_{m}^{3}}{3}\right]+\sum_{t,c,l,m,w}x_{clmwt}\times \left[\frac{CF_{m}^{1}+CF_{m}^{2}+CF_{m}^{3}}{3}\right]\\ +\sum_{t,p,r,c,l,m,w=1}u_{prclmwt}\times \left[\frac{\omega_{prm}^{1}+\omega_{prm}^{2}+\omega_{prm}^{3}}{3}\right]\times \left[\frac{CV_{m}^{1}+CV_{m}^{2}+CV_{m}^{3}}{3}\right]+\\ \sum_{t,m}N_{mt}\times CB_{m}+\sum_{t=2,l,m}x_{clmwt}\times \left[\frac{CINS_{m}^{1}+CINS_{m}^{2}+CINS_{m}^{3}}{3}\right]\\ +\sum_{t,p}I_{pt}\times \left[\frac{CH_{p}^{1}+CH_{p}^{2}+CH_{p}^{3}}{3}\right]+\sum_{w,t}NO_{wt}\times \left[\frac{CO_{w}^{1}+CO_{w}^{2}+CO_{w}^{3}}{3}\right], \end{array}$$

(4) $$\begin{array}{c} \text{Min }Z_{2}=\sum_{t,p,r,c,l,m,w}u_{prclmwt}\times g_{pm}, \end{array}$$

(5) $$\begin{array}{c} \text{Min }Z_{3}=\sum_{p,t}S_{pt}\;, \end{array}$$

(6) $$\begin{array}{c} u_{prclmwt}\leq y_{prmw}*\;x_{clmwt}*\left[\left(2\alpha -1\right)d_{pt}^{\left(1\right)}+\left(2-2\alpha \right)d_{pt}^{\left(2\right)}\right]\\ \forall \;p,r=\left\{1,\ldots ,R_{p}\right\},c,l,m,w,t, \end{array}$$

(7) $$\begin{array}{c} \sum_{c,l,m,w}u_{prclmwt}+I_{p,t-1}+S_{pt}-I_{pt}=\left[\left(2\alpha -1\right)d_{pt}^{\left(3\right)}+\left(2-2\alpha \right)d_{pt}^{\left(2\right)}\right]\;\forall p,\;r=R_{p},t, \end{array}$$

(8) $$\begin{array}{c} \sum_{l,m,w}x_{clmwt}\times A_{m}\leq Ucap_{c}\forall c,t, \end{array}$$

(9) $$\begin{array}{c} \sum_{l,m,w}x_{clmwt}\times A_{m}\leq Ucap_{c}\forall c,t. \end{array}$$

(10) $$\begin{array}{c} \sum_{l,m,w}x_{clmwt}\times A_{m}\geq Lcap_{c}\forall c,t, \end{array}$$

(11) $$\begin{array}{c} \sum_{p,r,c,w}u_{prclmwt}\times \omega_{prm}\leq \left[\left(2\alpha -1\right){\text{cap}}_{m}^{\left(1\right)}+\left(2-2\alpha \right){\text{cap}}_{m}^{\left(2\right)}\right]\forall l,m,t, \end{array}$$

(12) $$\begin{array}{c} \sum_{c,l,m,w}NP_{prc\overset{´}{c}l\overset{´}{l}m\overset{´}{m}t}=u_{p,r+1,\overset{´}{c}\overset{´}{l}\overset{´}{m}\overset{´}{w}t}\;\forall p,\overset{´}{c},\overset{´}{l},\overset{´}{m},\overset{´}{w},t,r=1,\ldots ,R_{p}-1, \end{array}$$

(13) $$\begin{array}{c} \sum_{\overset{´}{l},\overset{´}{c},\overset{´}{m},\overset{´}{w}}NP_{prc\overset{´}{c}l\overset{´}{l}m\overset{´}{m}t}=u_{prclmwt}\;\forall p,c,l,m,w,t,r, \end{array}$$

(14) $$\begin{array}{c} \sum_{c,l,w}x_{clmw,t+1}-\sum_{c,l,w}x_{clmwt}=N_{m,t+1}\;\forall m,t=1,\ldots ,T-1\;v, \end{array}$$

(15) $$\begin{array}{c} \sum_{c,l,w}x_{clmw,1}=N_{m,1}\;\forall m, \end{array}$$

(16) $$\begin{array}{c} \sum_{c,l,w}x_{clmw,1}=NO_{wt}\forall w,t, \end{array}$$

(17) $$\begin{array}{c} \left(1-\sum_{c,w}x_{clmwt}\right)\times \sum_{c,w}x_{clmw,t+1}=v_{lm,t+1}\;\forall l,m,t=1,\ldots ,T-1, \end{array}$$

(18) $$\begin{array}{c} \sum_{c,w}x_{clmwt}\times \left(1-\sum_{c,w}x_{clmwt,t+1}\right)=d_{lm,t+1}\;\forall l,m,t=1,\ldots ,T-1, \end{array}$$

(19) $$\begin{array}{c} \sum_{p}I_{pt}\times V_{p}\leq \left[\left(2\alpha -1\right)T{\text{cap}}^{\left(1\right)}+\left(2-2\alpha \right)T{\text{cap}}^{\left(2\right)}\right]\forall t, \end{array}$$

(20) $$\begin{array}{c} \sum_{p,r,c,l,m,w}u_{prclmwt}\times g_{pm}\leq G\text{cap}\;\forall t, \end{array}$$

(21) $$\begin{array}{c} I_{p,0}=0\;\forall p, \end{array}$$

(22) $$\begin{array}{c} \sum_{p}S_{pt}\leq SS\;\forall t, \end{array}$$

(23) $$\begin{array}{c} d_{lmt},v_{lmt},y_{prmw},x_{clmwt}=\left\{0,1\right\},\\ S_{pt},I_{pt},u_{prclmtw},NP_{prc\overset{´}{c}l\overset{´}{l}m\overset{´}{m}t},N_{mt},NO_{wt}\geq 0. \end{array}$$

Relation (3) is the first objective function that is to minimize costs. In fact, it is considered as an economic objective function that each component is the cost of transferring products between cells, the cost of transferring products inside cells, the cost of installing machines, the cost of removing machines, the overhead cost of machines, the variable cost of machines per operating time, the cost of purchasing machines, the cost of installing machinery in the first period, the cost of maintaining products in the workshop warehouse, and also the last component is the cost of the operator per period of time, respectively. Relation (4) is the second objective function that is to minimize the amount of carbon dioxide emissions by each type of machine per unit of product manufacturing. It is also introduced as an environmental objective function. Relation (5) as the third objective function is to minimize the shortage of estimation of customer demand, which conveys the concept of increasing customer satisfaction, and is called the objective function of social responsibility.

Relation (6) states that products can be manufactured by the relevant machines and operators to the maximum of the demand in each period. There must also be the ability to manufacture the desired product by the machine and the operator assigned. Relation (7) shows the inventory level of the workshop warehouse as well as the lost opportunity to supply the product (shortage) at any time for each type of product. In fact, it states that the amount of product manufactured in each time period plus the shortage and inventory of the previous period must be equal to the amount of demand plus inventory of this period. Relation (8) shows that each place in each time period can be assigned to a maximum of one machine. Relation (9) shows the maximum capacity of the cell for the placement of machines. Relation (10) shows the minimum number of machines to be placed in each given cell. Relation (11) indicates the time capacity of each machine in each time period to manufacture a product. In other words, each product needs an amount of time to manufacture, and this relation indicates the allowable amount of using each machine in each time period. Relations (12) and (13) are the constraints of maintaining the flow. Relation (14) shows the number of machines added to the workshop level in time periods. In fact, it shows the number of machines purchased in each period. Relation (15) shows the number of machines purchased in the first time period. Relation (16) shows the number of operators in time periods. Relations (17) and (18) show the amount of movement of machines or, in fact, the installation and removal of machines in each time period. Relation (19) shows the warehouse capacity of the workshop to store products after manufacturing. Relation (20) states the allowable amount of Co_2_ emissions. Relation (21) indicates that the warehouse is empty on day zero. Relation (22) states that the shortages must not exceed a certain amount.

It should be noted that the constraints related to Relations (17) and (18) are nonlinear and are linearized as follows (Bayram and Sahin [30]):

(24) $$\begin{array}{c} 0.5+v_{lm,t+1}+\sum_{c,w}x_{clmwt}-\sum_{c,w}x_{clmw,t+1}\geq 0\;\forall l,m,t=1,\ldots ,T-1,\\ 1.5+v_{lm,t+1}+\sum_{c,w}x_{clmw,t+1}-\sum_{c,w}x_{clmw,t+1}-1\leq 0\;\forall l,m,t=1,\ldots ,T-1,\\ 0.5+d_{lm,t+1}+\sum_{c,w}x_{clmwt}-\sum_{c,w}x_{clmwt}\geq 0\;\forall l,m,t=1,\ldots ,T-1,\\ 1.5+d_{lm,t+1}+\sum_{c,w}x_{clmwt}-\sum_{c,w}x_{clmw,t+1}-1\leq 0\;\forall l,m,t=1,\ldots ,T-1. \end{array}$$

There is no variable change in the linearization performed by the base paper, and the linearization is performed as presented above.

## 4. Solving the Problem and Providing the Results

### 4.1. Validation of the Proposed Model

In order to validate the proposed model, an example with small dimensions and its decision variables are examined to confirm the accuracy of the model. Due to the multipurpose nature of the proposed model, the augmented epsilon constraint method in GAMS software is used to provide the Pareto front related to the three objective functions of this model. The number of product types is 2, the number of operations is 3, the number of cells is 2, the number of places for machines is 5, the number of types of machines is 2, and the type of expertise of operators is 3. And this manufacturing of products is done in 4 days. The results of solving the proposed problem by GAMS software and the AEC method and the Pareto front formed are shown in the next steps.

Based on the two-dimensional Pareto front formed by the AEC method, it can be concluded that each of the two and three objective functions is in conflict with the first objective function, meaning that with increasing costs separately, carbon dioxide emissions are reduced, and the shortages of meeting the demand are also decreased. In this section, the optimal point for analysis is selected as follows: (cost = 567451, environment = 23014, social responsibility = 172). Also, GAMS software can solve problems 1 to 4 (Table 2), and subsequent problems are almost unsolvable due to a significant increase in solution time, and this issue, as well as the expression of the complexity of this problem by other authors is proof that this problem is NP-Hard.

### 4.2. Solving the Problem with Meta-Heuristic Algorithms

After solving the problem by GAMS software, due to the NP-Hard nature of the proposed model, meta-heuristic algorithms are used. NSGA-II and MOGWO algorithms are used to solve this model. Then, to compare the two algorithms, 10 problems are designed, and each problem is executed by the algorithms 10 times. The algorithms are compared based on 5 indicators. First, the problem solved in the previous chapter is solved by the two proposed algorithms, and the Pareto front of all three methods is shown. It is then represented by *α*s with different values of change in objective functions. Then, the two algorithms are compared with each other by SNS, RAS, MID, Time, and Diversity metrics, and the results are presented by statistical analysis in Minitab software. To compare the algorithms based on 5 metrics: SNS, RAS, Diversity, MID, and Time, 10 problems are designed; the problem information is presented in Table 3. It should be noted that GAMS software has the ability to solve problems 1 to 4, and in solving the next problems, the solution time increases significantly, and they cannot be solved. Both this issue and the fact that other papers have found this problem to be complex is proof that this problem is NP-Hard.

**Figure 1:**
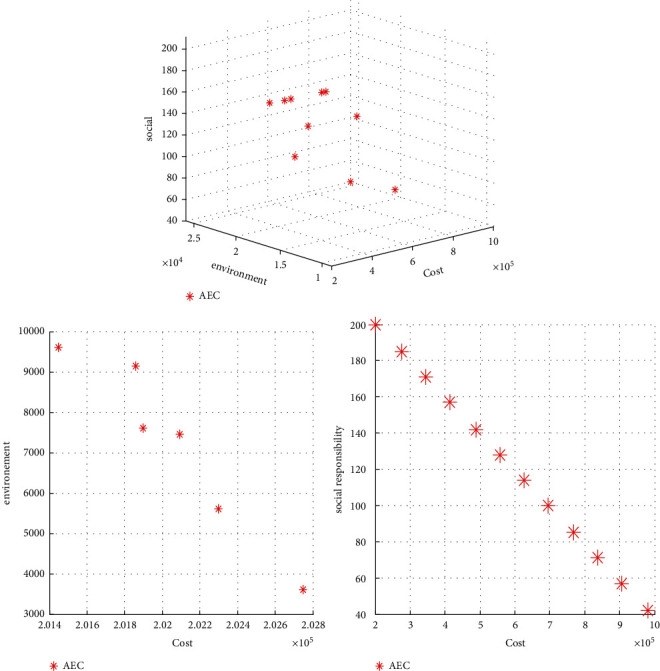
Three-dimensional and two-dimensional Pareto fronts resulting from problem solving for model validation.

**Figure 2:**
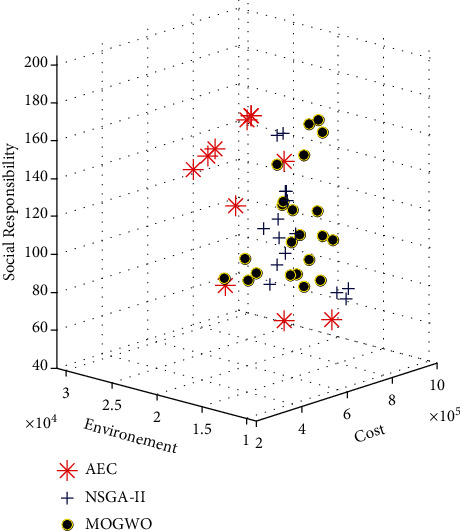
Pareto front formed by two methods of solving the small problem.

The problem is solved by the two proposed algorithms, and the Pareto front formed is shown in Figure 1. Then, a sensitivity analysis on *α* is carried out on the objective functions. The results are presented in Figure 2.

According to Figure 2, the algorithms have generated a Pareto front close to the Pareto front of the exact method, which is the augmented epsilon constraint (AEC). For each answer to the problem, this string is mapped to the problem variables. The output of the gray wolf algorithm in Table 4.

The output of the genetic algorithm is also Table 5.

**Figure 3:**
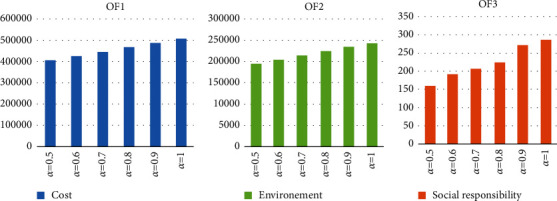
Sensitivity analysis of *α*.

As shown in Figure 3, with increasing *α*, due to the increasing effect on the pessimistic value of fuzzy parameters and the nature of minimizing the objective functions of these functions, their values increase. This means that with increasing demand for products and increasing costs of installation and purchase of machinery and fixed and variable manufacturing costs, costs, carbon dioxide emissions, and shortages increase.

### 4.3. Parameter Adjustment the by Taguchi Method

In Taguchi method, the factors affecting the test result are divided into two categories: uncontrollable (so-called noise (*N*)) and controllable (so-called signal (*S*)). An *S*/*N* variable is then defined, which is the signal-to-noise ratio. Taguchi parameter setting method adjusts the factors to levels that maximize the *S*/*N* ratio. One of the meta-heuristic methods used in this research is NSGA-II algorithm, four parameters MaxIt, NPOP, PC, PM must be set at optimal levels. For this purpose, first, three levels of low (1), medium (2), and high (3) are defined separately for each parameter to solve the problems, which are given in Table 6. Then, the set of experiments proposed by Taguchi method is calculated for four factors in three levels, in which 9 different modes are designed by Taguchi method. (It should be noted that each experiment is performed 10 times and their average is recorded, which is done to reduce the error of the algorithms and the answer will be more reliable.)

Now, in order to create an output from each experiment using the following method, all the indicators will be converted into one answer. First, the nature of each indicator based on negativity (less - better) or positivity (more - better) must be determined, in which case, the more the Diversity index, the better, and other indicators are negative in nature.  Table 7 shows the different indicators and positions that are the same designed experiments defined by *O*_*i*_.where *x*_*j*_ and *Q*_*i*_ represent the *j*-th criterion and the *i*-th position, respectively. Also, *r*_*ij*_ is equal to the value that position *i* in the indicator *j* will take. The obtained values of the indicators are normalized according to the fuzzy unscaling technique in Relations.

(25) $$\begin{array}{c} x_{j}^{+}⟶R_{ij}=\frac{r_{ij}-\min\left(r_{ij}\right)}{\max\left(r_{ij}\right)-\min\left(r_{ij}\right)}, \end{array}$$

(26) $$\begin{array}{c} x_{j}^{-}⟶R_{ij}=\frac{\max\left(r_{ij}\right)-r_{ij}}{\max\left(r_{ij}\right)-\min\left(r_{ij}\right)}. \end{array}$$

In this method of normalization, indicators with a negative nature will have a positive nature. Using the goal programming approach, the indicators are prioritized in terms of importance and the weight for each indicator is considered accordingly. In this research, the importance of each weight is applied according to Rabbani et al. [8]. Then, according to the significance coefficients, the total weight of the indicators of each experiment is calculated in the *Response* equation.

(27) $$\begin{array}{c} \text{Response}=\sqrt[{2}]{{\left(MI\;D\right)}^{2}+{\left(RAS\right)}^{2}+{\left(SNS\right)}^{1}+{\left(SNS\right)}^{1}+{\left(\text{Diversity}\right)}^{1}+{\left(\text{Spacing}\right)}^{1}}. \end{array}$$

Now, based on the response values of each algorithm, the *S*/*N* rate is calculated and based on it, the levels of each input parameter are determined. To do this, the resulting response is presented as the final response to the experiments, and the Taguchi more-better formula is used to calculate the S/N ratio. The results of the algorithms are given in Tables 8–12, respectively.

### 4.4. Evaluation Indicators

If a single-objective optimization problem *Z*_1_ minimization problem) is considered, it is clear that any solution that offers a possible solution for which *Z*_1_ is less is better. But in the case of the multiobjective decision-making (MODM) problem, the evaluation method is different, and it cannot be evaluated as single objective. In general, in evaluating MODM solution methods, it is important to note that each method has better performance with the criterion of greater maximum diversity, the criterion of less distance from the ideal solution (MID), the less value of RAS and the criterion of spacing, and more SNS. Accordingly, we define the following criteria for evaluating the two MODM methods and comparing them with each other.

The criterion of mean deviations from the ideal solution (MID criterion): in this criterion mentioned by Coello et al. (Coello, Lamont and Veldhuizen [31]) in 2007, first, an ideal answer to the problem is considered and then the mean deviation of Pareto set of solutions is calculated from the ideal solution. The ideal solution, which is represented by symbol *I*_sol_, is a mode in which both solutions are in their optimal state simultaneously *I*_sol_=(min(*z*_1_), min(*z*_2_)), or in minimization problems, the origin of the coordinates can be considered as the ideal solution (*I*_sol_=(0,0)). If *F*(*A*) represents the Pareto front of the solution method *A*, then the MID criterion is calculated as follows: where ‖*I*_sol_ − *pa*‖_2_ represents the Euclidean distance of the solution *pa ℇ F(A)* from the ideal solution. It is clear that the lower the MID criterion, the better (Behnamian et al. [32]).

(28) $$\begin{array}{c} MI\;D\left(A\right)=\frac{\sum_{pa\in F\left(A\right)}{\left\|pa-I_{\mathrm{sol}}\right\|}_{2}}{\left\|F\left(A\right)\right\|}. \end{array}$$

Maximum diversity criterion: it measures the length of the space cube diameter used by the end values of the objectives for the set of nondominated solutions, which is calculated in the following equation. The larger the value, the better [8].

(29) $$\begin{array}{c} \text{Diversity}=\sqrt{{\left(\text{max}f_{1i}-\text{min}f_{1i}\right)}^{2}+{\left(\text{max}f_{2i}-\text{min}f_{2i}\right)}^{2}}. \end{array}$$

Uniformity or spacing criterion: it calculates the relative distance of consecutive solutions using the following equation. The smaller the spacing, the better (Schott [33]) (Kaveh and Mahdavi [34]).

(30) $$\begin{array}{c} \text{Spacing}={\left(\frac{1}{n_{pf}}\overset{n_{pf}}{\underset{i=1}{\sum }}{\left(d_{i}-\bar{d}\right)}^{2}\right)}^{1/2},\text{where }\bar{d}=\frac{1}{n_{pf}}\sum_{i=1}^{n_{pf}}d_{i}. \end{array}$$

Criteria of the spread of nondominated solutions (SNS criterion): this indicator can be expressed as an indicator of diversity as follows:

(31) $$\begin{array}{c} \mathrm{SNS}=\sqrt{\frac{\sum_{i=1}^{n}{\left(\mathrm{MID}-C_{i}\right)}^{2}}{n-1}}. \end{array}$$

The higher the SNS value, the better the algorithm. Criterion of rate of achievement to the two objectives simultaneously (RAS): another evaluation criterion proposed in this research is RAS. The RAS equation is shownas follows:

(32) $$\begin{array}{c} \mathrm{RAS}=\frac{\sum_{i=1}^{n}\left(f_{1i}-F_{i}/F_{i}\right)+\left(f_{2i}-F_{i}/F_{i}\right)}{n}. \end{array}$$

In Relation (32), *F*_*i*_ = min{*f*_1*i*_, *f*_2*i*_}. The lower the RAS value, the better. It also considers positive objective value in all indicators; therefore, negative objectives must be turned into the positive part of the axis. Each problem is now implemented 10 times for each algorithm, and the graph of each indicator for the two MOGWO and NSGA-II methods is shown below.

**Figure 4:**
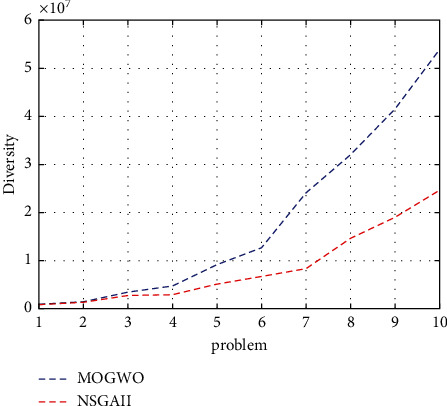
Diversity indicator for the research algorithms.

The indicator we are examining is the diversity indicator.  Figure 4 shows the graph of each algorithm as a comparison diagram of both algorithms according to this indicator. Considering the shape and nature of this indicator, the higher it is, the better. It is clear that the MOGWO algorithm performed better than the NSGA-II algorithm.

**Figure 5:**
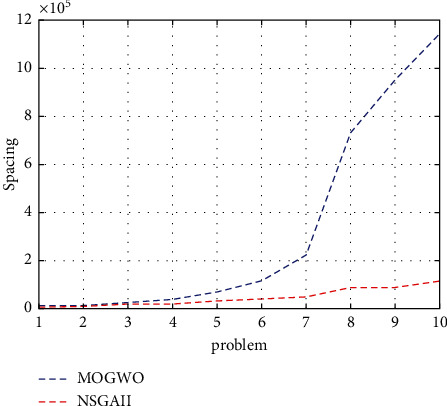
MID indicator for the research algorithms.

For the MID indicator, any algorithm that has a smaller value performs better. According to Figure 5, the MOGWO algorithm is always better than the NSGA-I.

**Figure 6:**
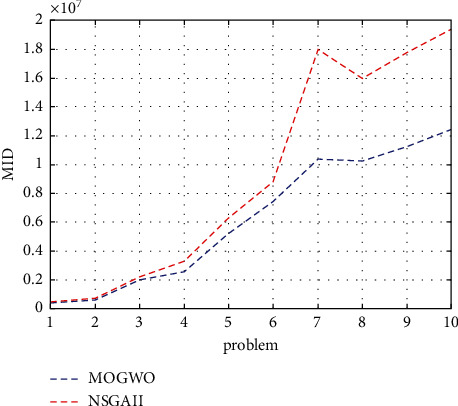
Spacing indicator for the research algorithms.

In terms of Spacing indicator, it can be said that the NSGA-II algorithm performed better than the MOGWO algorithm, which is shown in Figure 6.

**Figure 7:**
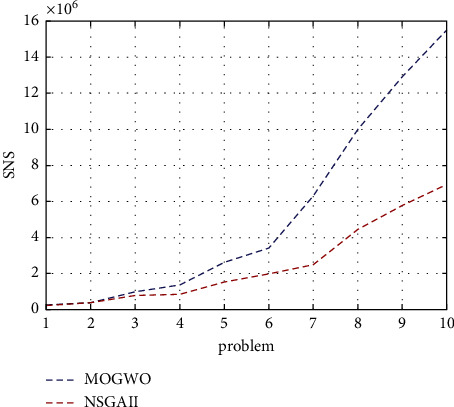
SNS indicator for the research algorithms.

**Figure 8:**
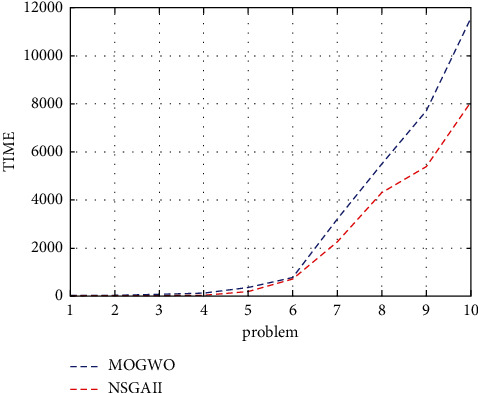
Time indicator for the research algorithms.

The higher the SNS indicator, the better. According to Figures 7 and 8, it can be concluded that the MOGWO algorithm is better than the NSGA-II.

**Figure 9:**
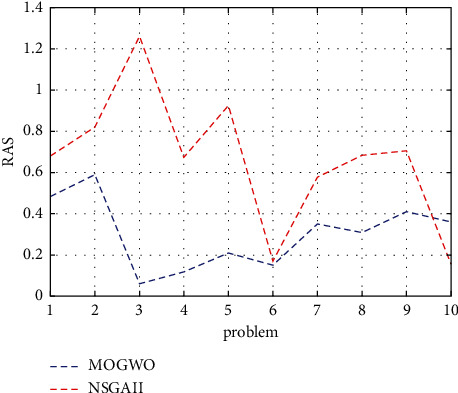
RAS indicator for the research algorithms.

The last indicator studied in this research is the rate of achievement to the two objectives simultaneously (RAS) to solution, which, as mentioned earlier, has a negative nature (the less - the better), i.e. any algorithm that has a lower RAS value is better. According to Figure 9, it is clear that the MOGWO algorithm performed better.

## 5. Conclusion

In this research, a three-objective mathematical model was stated, the first of which was economic (cost), the second was environmental (air pollution), and the third was social (responsibility). All three objectives were minimization. The model had 14 assumptions and 17 constraints, and the demand for products was fuzzy. The model in the example taken from the real world of manufacturing (manufacturing of safe scaffolding that has all three dimensions of sustainable manufacturing) was solved by GAMZ software through the exact augmented epsilon constraint method (AEC). Due to the fact that the model is NP-hard, meta-heuristic algorithms were used to solve the model. Due to the high complexity of the problem, two algorithms, NSGA-II and MOGWO, were used to solve the proposed model. Finally, 10 problems with different dimensions were solved to evaluate the performance of these two algorithms in MATLAB software and the performance of these two algorithms was determined. The output of the algorithms has produced Pareto fronts close to the Pareto front of the exact method, which is the augmented epsilon constraint (AEC). Sensitivity analysis of *α* was carried out, and we increased the alpha value step by step. As *α* increases due to the effect on the pessimistic value of the fuzzy parameters and the nature of the minimization of the objective functions, these functions increase in value. This means that with increasing demand for products and increasing the cost of installation and purchase of machinery and fixed and variable production costs, costs, carbon dioxide emissions, and shortages increase. Finally, we carried out statistical analysis and comparison of two meta-heuristic algorithms. First, four parameters of MaxIt, NPOP, PC, PM for two algorithms were set at optimal levels by adjusting the parameter by Taguchi method, and the results of experimental problems of five indicators were obtained and the output of the mini-tab software for adjusting the parameters of the two algorithms was shown by Taguchi method. The criterion of mean deviations from the ideal solutions (MID), maximum diversity indicator, uniformity indicator, spread of nondominated solutions indicator, and the achievement of two objective functions simultaneously were the five indicators through which we compared the two algorithms. Ten problems were designed to compare algorithms. Then, each problem was implemented ten times for each algorithm, and the average was reported as the final answer for each solution method, which showed the superiority of the multiobjective gray wolf algorithm.

Social, economic, and environmental dimensions each have thousands of criteria. As a result, there is a lot of space for conducting research in this field. The concepts of sustainable production are the newest concepts in manufacturing systems and are not limited to manufacturing industries, and research in this field conducted in recent years confirms this important point, as noted in the review of the research literature, developed industrialized countries have made these concepts binding in all public and private departments and monitor their proper implementation. It is suggested that an institution be established in our country or that there be a department in every organization and manufacturing unit to help institutionalize these concepts in the country while researching in this field and stating the required instructions. Existence of different manufacturing methods can include the dimensions of sustainable production in them, and the benefits of sustainable production can be seen in them. Other meta-heuristic methods can be used to solve the model.

## Figures and Tables

**Table 1:** Literature review of sustainable manufacturing dimensions.

Social dimension	Economy	Salary and benefits	[12]
	Satisfaction level	Level of employee satisfaction, customer churn	[6]
	Quality and health	Employee health and safety programs, human factor engineering, average travel distance of employees to the company	[18]
			[7]
	Human resources and society	Workforce access, workforce skills, workforce selection, employee learning hours, employee performance appraisal, society development	[11]
			[12]

Environmental dimension	Environmental management	Environmental standards, environmental characteristics and goals, structure responsible for the environment, environmental assessment	[7]
			[8]
			[24]
	Environmental aspects, responsibility	Relationship between suppliers and the environment, the company's view of the environment, the type of waste treatment, the consumption of hazardous substances	[17]
			[1]
	Consumption	Water, energy, and paper	[23]

Economic dimension	Economic management	Market distribution, number of recycled materials	[22]
	Operational efficiency and products	Life cycle, lean manufacturing waste, start-up time, flexibility, inventory, quality of products and services, society quality management, new products, initiative in international markets, design for production and assembly	[13]
	Operational results and suppliers	Profit, price and operational indicators, standard for suppliers, delivery	[27]
			[8]
	Customers	Number of customer complaints about the area, delivery time	[22]
			[28]
	Infrastructure	Proximity to transportation centers, hubs, access to alternative transportation, access to storage facilities, efficiency of using transportation resources, access to manufacturing facilities	[17]
			[1]
			[8]

**Table 2:** EC output from GAMS.

Problem	EC					
	Time	Diversity	Spacing	Mid	SNS	RAS
3 ∗ 2 ∗ 2 ∗ 2 ∗ 2 ∗ 2 ∗ 2	23.256	897225	7411	214558	147226	0.24755
3 ∗ 2 ∗ 2 ∗ 3 ∗ 2 ∗ 2 ∗ 2	88.256	1036685	11478	474558	478226	0.14778
3 ∗ 3 ∗ 3 ∗ 4 ∗ 3 ∗ 3 ∗ 3	178.669	1255369	18996	741058	1455230	0.01477
3 ∗ 3 ∗ 3 ∗ 5 ∗ 3 ∗ 3 ∗ 3	879.614	2476698	29880	1011475	1854701	0.08958

**Table 3:** Information on 10 numerical problems.

Problem dimensions (*P* ∗ *R* ∗ *C* ∗ *L* ∗ *M* ∗ *W* ∗ *H*)	Uniform data	Parameter	Uniform data	Parameter
3 ∗ 2 ∗ 2 ∗ 2 ∗ 2 ∗ 2 ∗ 2	[5, 7]	*ω*_*prm*_	[70, 50]	*d*_*ph*_
3 ∗ 2 ∗ 2 ∗ 3 ∗ 2 ∗ 2 ∗ 2	[750, 650]	cap_*m*_	[80, 60]	*I*_*p*_
3 ∗ 3 ∗ 3 ∗ 4 ∗ 3 ∗ 3 ∗ 3	[1, 0]	*δ*_*prmw*_	[150, 110]	*E*_*p*_
3 ∗ 3 ∗ 3 ∗ 5 ∗ 3 ∗ 3 ∗ 3	[1300, 1100]	*T*cap	[60, 40]	*Q*_*ll*′_
4 ∗ 4 ∗ 4 ∗ 6 ∗ 4 ∗ 4 ∗ 4	[1800, 1600]	*G*cap	[500, 450]	*μ*_*m*_
5 ∗ 5 ∗ 5 ∗ 7 ∗ 5 ∗ 5 ∗ 5	[17, 12]	*V*_*p*_	[300, 250]	*π*_*m*_
5 ∗ 5 ∗ 5 ∗ 8 ∗ 5 ∗ 5 ∗ 5	[160, 120]	*OC*_*w*_	[110, 70]	*FC*_*m*_
6 ∗ 6 ∗ 6 ∗ 10 ∗ 6 ∗ 6 ∗ 6	[70, 30]	*g*_*pm*_	[70, 30]	*VC*_*m*_
6 ∗ 6 ∗ 6 ∗ 12 ∗ 6 ∗ 6 ∗ 6	[50, 30]	*HC*_*p*_	[450, 270]	*φ*_*m*_
7 ∗ 7 ∗ 7 ∗ 15 ∗ 7 ∗ 7 ∗ 7	[3, 2]	*A*_*m*_	[6, 5]	*U*cap_*c*_
			[4, 2]	*L*cap_*c*_

**Table 4:** Output of gray wolf algorithm.

The first objective function	174580.39
The second objective function	4750
Third objective function	257
Sum *u*_*prclmhw*_	145
Sum *U*_*prcc*′*ll*′*mm*´*h*_	65
Sum of deficiency	245
Deficit rate *S*_*ph*_	11 13 5 6 0
	8 14 4 5 0
Inventory amount *I*_*ph*_	0 15 5 17 0
	0 26 25 14 0

**Table 5:** Output of the genetic algorithm.

The first objective function	165227.58
The second objective function	5620
Third objective function	320
Sum *u*_*prclmhw*_	92
Sum *U*_*prcc*′*ll*′*mm*´*h*_	65
Sum of deficiency	285
Deficit rate *S*_*ph*_	8 20 15 1 0
	4 5 7 1 0
Inventory amount *I*_*ph*_	0 16 14 1 0
	0 25 12 25 1

**Table 6:** Defined levels for the genetic algorithm parameters to solve problems.

GA parameter/factor	Low level (1)	Intermediate level (2)	High level (3)
Max it	100	150	200
Npop	50	75	100
PC	0.7	0.8	0.9
PM	0.2	0.3	0.4

**Table 7:** Nature of the research indicators.

*Q*_*i*_	+	−	+	−
	*x*_1_(diversity)	*x*_2_(*MI* *D*)	*x*_3_ (*SNS*)	*x*_4_(*RAS*)
*Q*_1_	*r*_11_	*r*_12_	*r*_13_	*r*_14_
*Q*_2_	*r*_21_	*r*_22_	*r*_23_	*r*_24_
*Q*_*i* _	*r*_*ij*_	*r*_*ij*_	*r*_*ij*_	*r*_*ij*_

**Table 8:** Response values for the NSGA-II algorithm.

Response	1.6592	2.1457	1.2587	1.3624	1.5147	1.1478	1.5214	1.6985	1.4787

**Table 9:** Result of setting the parameters of NSGA-II algorithm by Taguchi method.

PM	PC	Npop	Max it
0.3	0.8	75	100

**Table 10:** Defined levels for MOGWO algorithm parameters to solve problems.

GA parameter/factor	Low level (1)	Intermediate level (2)	High level (3)
Max it	100	150	200
Greywolves	50	100	150
A	0.5	0.75	1

**Table 11:** Response values for the MOGWO algorithm.

Response	1.3201	1.6515	2.1475	1.8711	2.1445	1.9874	2.1142	1.5446	2.1014

**Table 12:** Result of setting parameters of MOGWO algorithm by Taguchi method.

Max it	Greywolves	A
150	100	0.75

## Data Availability

The data used are included in the article.
